# Nitric Oxide Releasing Hydrogel Nanoparticles Decreases Epithelial Cell Injuries Associated With Airway Reopening

**DOI:** 10.3389/fbioe.2020.579788

**Published:** 2021-01-05

**Authors:** Samar Shurbaji, Ibrahim M. El-Sherbiny, Maha Alser, Isra H. Ali, Haya Kordi, Ameena Al-Sadi, Anton Popelka, Fatiha Benslimane, Magdi Yacoub, Huseyin C. Yalcin

**Affiliations:** ^1^Biomedical Research Center, Qatar University, Doha, Qatar; ^2^Nanomedicine Lab, Center of Materials Sciences, Zewail City of Science and Technology, Giza, Egypt; ^3^Department of Biomedical Sciences, College of Health Science-QU Health, Qatar University, Doha, Qatar; ^4^Center for Advanced Materials, Qatar University, Doha, Qatar; ^5^Heart Science Centre, Imperial College, National Heart and Lung Institute, London, United Kingdom

**Keywords:** nitric oxide, hydrogel nanoparticle, acute respiratory distress syndrome, mechanical ventilation, COVID-19, SARS-CoV-2

## Abstract

Acute respiratory distress syndrome (ARDS) is an acute inflammatory lung condition. It is characterized by disruption of gas exchange inside the alveoli, accumulation of protein edema, and an increase in lung stiffness. One major cause of ARDS is a lung infection, such as SARS-COV-2 infection. Lungs of ARDS patients need to be mechanically ventilated for airway reopening. Consequently, ventilation might damage delicate lung tissue leading to excess edema, known as ventilator-induced lung injury (VILI). Mortality of COVID-19 patients under VILI seems to be higher than non-COVID patients, necessitating effective preventative therapies. VILI occurs when small air bubbles form in the alveoli, injuring epithelial cells (EPC) due to shear stress. Nitric oxide (NO) inhalation was suggested as a therapy for ARDS, however, it was shown that it is not effective because of the extremely short half-life of NO. In this study, NO-releasing nanoparticles were produced and tested in an *in vitro* model, representing airways in the deep lung. Cellular injuries were quantified via fluorescent live/dead assay. Atomic force microscopy (AFM) was used to assess cell morphology. qRT-PCR was performed to assess the expression of inflammatory markers, specifically IL6 and CCL2. ELISA was performed to assess IL6 and confirm qRT-PCR results at the protein level. Finally, ROS levels were assessed in all groups. Here, we show that NO delivery via nanoparticles enhanced EPC survival and recovery, AFM measurements revealed that NO exposure affect cell morphology, while qRT-PCR demonstrated a significant downregulation in IL6 and CCL2 expression when treating the cells to NO both before and after shear exposure. ELISA results for IL6 confirmed qRT-PCR data. ROS experiment results support our findings from previous experiments. These findings demonstrate that NO-releasing nanoparticles can be used as an effective delivery approach of NO to deep lung to prevent/reduce ARDS associated inflammation and cell injuries. This information is particularly useful to treat severe ARDS due to COVID-19 infection. These nanoparticles will be useful when clinically administrated to COVID-19 patients to reduce the symptoms originating from lung distress.

## Introduction

Acute respiratory distress syndrome (ARDS) is a clinical condition that is characterized by increased permeability of pulmonary/capillary barrier in the gas exchange sites in the lung, resulting in the accumulation of edema fluid and hypoxia in the lung (Koh, 2014). Presence of edema fluid results in the inactivation of the surfactant molecules in the thin liquid layer over the epithelial cells. In normal lungs, this layer acts to protect the cells by decreasing surface tension and in diseased lungs, as a result of inactivation of these molecules, epithelial cells become prone to injury. One of the major causes of ARDS is viral infections such as SARS-COV-2 (Pan et al., 2020; Yuki et al., 2020). ARDS is mainly managed by mechanical ventilation therapy that involves inducing positive pressure to the lungs, which in turn improves breathing and gas exchange. However, mechanical ventilation can induce overdistension of gas exchange sites resulting in propagation of small bubbles in terminal bronchioles and alveoli, resulting in exposure of shear stress to the epithelial cells (EpCs). These mechanical forces might induce further injury to the already diseased tissue, a phenomenon known as ventilator-induced lung injury (VILI) (Biehl et al., 2013).

Patients infected with the recent SARS-CoV-2 were shown to have severe pneumonia that is classified as ARDS (Yang et al., 2020). ARDS among COVID-19 patients is termed as CARDS (Marini and Gattinoni, 2020). It is hard to estimate the number of COVID-19 cases who died due to CARDS. Nevertheless, a meta-analysis was published in China, where seven clinical studies were analyzed and reported an estimation of CARDS ratio that was 19.5% and the death rate was around 5.5% (Zhu et al., 2020). Moreover, it has been reported that ARDS is a major cause of death in the Intensive Care Unit (ICU) patients (Ruan et al., 2020; Onder et al., 2020). There is a variation in reported data in different studies, however, ARDS was reported as a serious issue in all studies, which raises the problem of VILI as well. It was hypothesized by Tremblay and Slutsky in 1998 that VILI causes death due to biotrauma associated with cytokine storms (Tremblay and Slutsky, 1998). Blocking the cytokine storms has been suggested as a successful approach to treat ARDS due to COVID-19 (Zhang et al., 2020). Moreover, the mortality of mechanically ventilated COVID-19 patients is higher than non-COVID ARDS patients, making this patient sub-population particularly vulnerable to VILI (personnel communication with ICU clinicians treating COVID patients). Currently, there is no established pharmaceutical therapy of ARDS or VILI, particularly for COVID-19 patients. New approaches are needed to minimize the damage caused by VILI which would lead to a decrease in mortality rates among mechanically ventilated ARDS and CARDS patients.

Nitric oxide (NO) inhalation was suggested as a potential therapy against VILI. NO is a free radical that plays an important role in many physiological processes (Friedman, 2015). NO is an endothelium-derived relaxing factor and a key mediator of vasodilation (Cockcroft, 2005). A decrease in NO production by endothelial cells was shown to result in arterial stiffening and hypertension (Hermann et al., 2006). NO was shown to work as a pulmonary vasodilator as well from studies in ARDS patients and direct inhalation improved ventilation-perfusion and oxygenation in these patients (Akmal and Hasan, 2008; Gebistorf et al., 2017). NO administration is suggested to be effective against lung inflammation as well (Tripathi et al., 2007). However, NO has an extremely short biological half-life (i.e., few seconds) preventing its clinical use in pulmonary diseases (Akmal and Hasan, 2008). Nanotechnology applications can potentially overcome this limitation by a combination of inhalation therapy with the use of a slow NO-releasing nanoparticle formulation. As such, our group has developed a new series of NO-nanomedicine formulations. The NO-releasing hydrogel nanocomposites-based nanoparticles (NO-RPs) were prepared via ionotropic gelation technique (Mohamed et al., 2016). The kinetics study carried out in an aqueous media showed that the NO released from the nanoparticles reached a peak in about 1 h. NO-RPs did not result in cell death or any inflammatory response when tested with cultured endothelial cells. Also, they induced concentration-dependent relaxations of both aorta and pulmonary arteries in mice, suggesting non-toxicity and beneficial potential effects in VILI. This indicates the ability of these nanoparticles to produce a stream of NO as a result of the reduction of nitrite salts incorporated within the particle structure. NO acts as a potent anti-proliferating vasodilating agent that will consequently cause the required relaxation in pulmonary arteries (Tonelli et al., 2013).

In the current study, we hypothesized that exposing NO-RPs to EpCs before mechanical ventilation will decrease inflammation and cellular injuries associated with mechanical ventilation. To test our hypothesis, we exposed rat L2 epithelial cells to airway reopening conditions using our *in vitro* flow perfusion experimental set up that we have developed previously (Yalcin et al., 2007; 2009). Before bubble propagations, cells were cultured with three differently formulated NO-RPs in independent experiments. All three NO-releasing nanoparticles are hydrogel nanocomposites-based formulations, and they were prepared through the ionotropic gelation technique. Different NO-RPs formulations resulted in different viabilities. In separate experiments, we exposed EpCs to bubble propagations which were followed by culture with NO-RPs. Again, we used three differently formulated nanoparticles in these experiments. Interestingly, when we kept the cells at cell media with NO-RPs following the bubble propagation, cell viabilities were enhanced, but to a lesser extent compared to exposure of particles before bubble propagations. Actin cytoskeleton immunocytochemistry showed actin fibers depolymerization for the cells cultured with the investigated NO-RPs. AFM cell topography measurements confirmed the decrease in cell volume consistent with Actin depolymerization. NO nanoparticle exposure both before and after the bubble exposure decreased expression levels of inflammatory markers such as IL6 and CCl2. Nanomedicine is a powerful approach for the delivery of active agents to pulmonary airways and alveoli, in the prevention of ARDS and VILI. Our results provide clear evidence that an efficient NO exposure strategy is a potentially beneficial approach for decreasing VILI for ARDS, in conditions such as severe SARS-CoV-2 infections.

## Materials and Methods

### Materials for NO-RPs

Sodium nitrite, L-cysteine, tetramethylorthosilicate, low molecular weight chitosan, ethanolamine, ferrous chloride (FeCl_2_), ferric chloride (FeCl_3_) anhydrous, sodium triphosphate, and hydrochloric acid were purchased from Sigma-Aldrich (Germany). All other solvents and reagents were of high purity and used as received.

### Synthesis of SPIONS

Appropriate amounts of FeCl_2_ and FeCl_3_ (molar ratio Fe^2+^: Fe^3+^ of 1:2) were dissolved in 20 ml of de-oxygenated bi-distilled water. Then, ethanolamine was used as the base, where ethanolamine solution (2.5%) was added dropwise to the reaction mixture under a nitrogen atmosphere with stirring at 60^*o*^C along with increasing the pH to 11. After 2 h of agitation, the resulting SPIONS were separated using an external magnet, washed with bi-distilled water, and dried under vacuum at 40^*o*^C.

### Characterization of SPIONS and the Developed NO-RPs

The size, polydispersity index, and the zeta potential of the different prepared NO-releasing nanoparticles (NO-RPs 1, 2, and 3) were obtained using Zetasizer (Nano-ZS, Malvern, United Kingdom). Morphology of SPIONS and NO-RPs was investigated using HR-TEM (JEM-2100F; JEOL, United States) and SEM (Nona Nano SEM, FEI, United States), respectively. The magnetic characteristics of the prepared SPIONS were measured using a vibrating sample magnetometer (VSM). The NO release pattern from the developed NO-RPs was assessed using a NO electrode.

### Cell Culture

Rat L2 EpCs (CCL-149, American Type Culture Collection, Manassas, VA) were used in this study (passage number less than 30). Cells were kept in Ham’s F-12K medium with 10% fetal bovine serum and 1% antibiotic-anti mycotic solution. The cell culture media was changed every 2–3 days. For each experiment, cells were harvested with 0.125% trypsin (Invitrogen), counted, and seeded onto 40-mm-diameter coverslips and placed in 60 mm diameter Petri dishes. 40 mm coverslips fit the bottom of the Bioptechs flow chamber and hence were used in this study (explained below). These coverslips were coated with (25 μl/ml) collagen solution to prevent cellular detachment during bubble exposure. A concentration of 8 × 10^4^ cell/ml and 2 ml cell media were prepared for each well, and cells were kept under suitable culture conditions (temperature: 37°C and 5% CO_2_) until confluence (around 4 days).

### Generation of Airway Reopening Conditions

**FIGURE 1:**
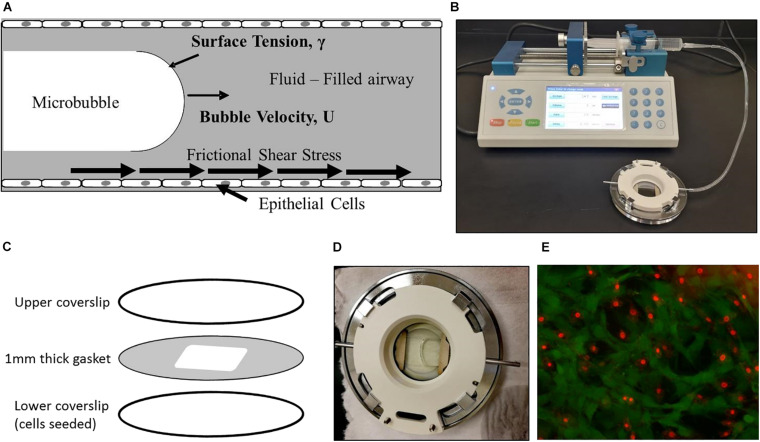
Experimental setup. **(A)** We expose epithelial cells to frictional shear stress relevant to airway reopening in our setup. **(B)** A single bubble is propagated over cell monolayer by filling the chamber and then retracting fluid over the cells. **(C)** The parallel plate flow chamber is composed of an upper coverslip, cell-seeded lower coverslip, and membrane sandwiched between two coverslips. **(D)** A propagating bubble is seen. **(E)** Fluorescent live/dead stain method used to assess viability. Here, green is calcein stained live cells, and red is ethidium stained dead cells.

In this study, we exposed lung EpCs to flow-induced stresses associated with airway reopening in ARDS (Figure 1A), to test the effect of NO exposure in decreasing cellular injuries during airway reopening. For this purpose, reopening conditions were generated using a parallel plate flow chamber (Bioptechs-FCS2 chamber, Figure 1B). The chamber consists of an upper coverslip, lower coverslip seeded with cells, and a silicone gasket sandwiched between the two coverslips. Silicon gasket thickness was selected to be 1 mm, to represent airways in the distal lung having 1 mm diameter (Yalcin et al., 2007, 2018; Figure 1C). Bubble propagation of 3 mm/s was generated over cell monolayers based on our flow calculations in the distal lung. PBS was used as the occlusion fluid since PBS represents a surfactant-deficient, high-surface tension airway-lining fluid, a characteristic of ARDS (Yalcin et al., 2018). Airway reopening was generated as follows: The flow chamber was first filled with PBS using the syringe at a rate of 3 mm/s, and control studies demonstrated that this initial filling of the chamber (PBS infusion) does not result in cell necrosis. Airway reopening was then simulated by withdrawing the fluid from the chamber at a constant speed of 3 mm/s. This results in the propagation of a single, long air bubble over the surface of the EpCs (Figure 1D). Once the air bubble had displaced the occlusion fluid, the chamber is refilled with a live/dead stain at 3 mm/s and incubated for 15 min before visualization under the fluorescent microscope (Figure 1E).

### NO Exposure and Experimental Groups

Three types of NO-releasing nanoparticles (NO-RPs) were used, those are NO-RPs1, NO-RPs2, and NO-RPs3. For these particles, peak NO release is at 1 h at aqueous media (Mohamed et al., 2016). We designed our 5 experimental groups accordingly, as follows: Control 1 (negative control) where we maintain cells in cell media at 37°C for 1 h; Control 2 (NO control) where we maintain cells in cell media with NO-RPs at 37°C for 1 h; Control 3 (positive control) where we expose cells to bubble propagation and then maintain in cell media at 37°C for 1 h; Control 4 where we treat cells with 0.5 μM Latrunculin A solution (latrunculin A is known to decrease the stiffness of the cells), Experimental group 1 (ExpGr1) where we maintain cells in cell media with NO-RPs at 37°C for 1 h followed by bubble propagation (survival group); and Experimental group 2 (ExpGr2) where we expose cells to bubble propagation and then maintain in cell media with NO-RPs at 37°C for 1 h (recovery group). For experiments involving NO-RPs, a concentration of 5 mg/ml was prepared by weighing NO-RPs and resuspending them in cell media. An amount of 2–3 ml of the suspension was added to each cell culture well. This concentration was suggested by Mohamed et al. (2016) as 5 mg/ml gave a peak NO concentration of about 200–300 nM.

### Viability Assessment

Cell viability after bubble propagation was determined by standard live/dead staining assay. The live/dead stain consisted of 1 μM ethidium homodimer 1 and 1.2 μM calcein-AM in serum-free media. After bubble propagations, cells were stained with 1 ml live/dead stain and were incubated in dark for 15 min. Cells were then visualized using a fluorescent microscope to be counted and analyzed. For each experiment, at least 10 fluorescent pictures were taken at 20X magnification and analyzed. Cells were counted by ImageJ software, where green is live cells, and red is dead cells (Figure 1E). The dead cell percentages were counted by dividing the number of dead cells by the total number of cells.

### Cytoskeletal Staining

Actin fibers are known as stress fibers as it gives mechanical strength to the cell. Those fibers can be stained using immunohistochemistry techniques. For immunostaining, CCL-149 L2 cells are grown on rectangular coverslip placed in wells of 6 well plate, with a concentration of 3 × 10^2^ cells/ml and 2 ml cell media. Cells were grown until confluency (around 4 days) with changing media every 2 days. The staining procedure started by washing the cells twice with 1X PBS and then fixing the cells with 4% paraformaldehyde for 15 min (2 ml in each well). After that, the cells were washed twice with PBS and permeabilized with 0.1% Triton X100 for 5 min. Then, the cells were blocked with 1% bovine serum albumin (BSA) for 15 min. The cells were then incubated with 2.5% Alexa-488 labeled phalloidin in the fridge (4 C°) overnight. Then, the cells were labeled with DAPI stain (0.002 mg/ml) for nuclei for 2 min and washed twice with distilled water. Following that, a drop of Gel Mount^TM^ was placed on a glass slide and the coverslip is placed over the Gel Mount^TM^ with cells facing down, the slides were then visualized with a fluorescent microscope. The blue filter is for Alexa-488 labeled Phalloidin, and the UV filter is for DAPI. In this study, we expected that when we expose cell monolayers to NO-RPs, actin fibers will be disrupted. To confirm changes in actin cytoskeletal structure in response to NO-RP exposure, we compared the cytoskeletal structure for NO-RP exposed cells with the positive control, Latrunculin (i.e., a known actin-depolymerizing agent) treated monolayers. NO-RP or LAtrunculin exposure was 1 h. Fluorescent pictures were taken under identical exposure settings to be able to compare the fluorescent intensity that will represent actin fiber density.

### Preparation of EpC Monolayers for Atomic Force Microscopy (AFM)

EpCs were grown on circular coverslip until confluence, and then treated with tested agents (i.e., 1 h No-RP exposure, 1 h Latrunculin exposure, or no treatment as the control group). Monolayers were then washed twice with PBS and fixed with 4% paraformaldehyde for 15 min. This was followed by one wash with PBS and the last wash with distilled water. After that, the slide was allowed to air dry and kept under protected conditions (Francis et al., 2010).

### Surface Topography Analysis With AFM

For surface topography analyses, an Atomic force microscope was used providing also information about surface roughness, represented by an average deviation (Ra) from the mean line over one sampling length. The AFM device MFP-3D (Asylum research, United States), equipped with a Silicon probe (Al reflex coated Veeco model—OLTESPA, Olympus) was used for measurement under ambient conditions using the Standard Topography AC air (tapping mode in the air) (Magdesian et al., 2012; Mikula et al., 2012; Noel et al., 2013).

### Relative Gene Expression of Inflammatory Markers

To verify the effect of NO on cells, qRT-qPCR was done to compare the relative gene expression level of different inflammatory factors released from rat EpC, including IL-6 and CCL2, and β-ACTIN as an endogenous control. Experiments with 3 biological replica tests were conducted on each experimental group. First, total RNA was extracted from the cells using the common TriZol extraction protocol (Qiagen #79306). Then, the total RNA was reverse transcribed into cDNA using the SuperScript IV VILO Reverse Transcriptase kit (Invitrogen 11756050) as recommended by the manufacturer. Primers were obtained from IDT (listed in Table 1) and used for the analysis. qRT-PCR was performed in three technical replicates for the three genes using the SYBR Green master mix (applied biosystems) as suggested by the manufacturer. The expression levels were normalized to the β-ACTIN expression level. The relative quantity was calculated using ΔΔC_*T*_ method described by Schmittgen and Livak (2008). The detailed methodology is presented in Supplementary Materials.

**TABLE 1:** Primer’s list.

Gene name	Code	Sequence
Beta-actin II	β-actin II	Fw: AGA TTA CTG CCC TGG CTC CTA G
		Rv: GAC TCA TCG TAC TCC TGC TTG C
Interleukin-6	IL6	Fw: GAC AAA GCC AGA GTC ATT CAG AG
		Rv: TTG GAT GGT CTT GGT CCT TAG CC
C-C motif	CCL2	Fw: ATG ATC CCA ATG AGT CGG CTG GAG
chemokine 2		Rv: GCA CAG ATC TCT CTC TTG AGC TTG G

### Rat IL-6 ELISA

The level of IL6 was measured through EpC L2 (ATCC^®^ CCL–149^TM^) cell supernatants using Quantikine ELISA Kit (R6000B; R&D Systems). The experiment was conducted according to the manufacturer’s instructions. Briefly, control and standards were prepared before loading the samples. 50 μl of assay diluent was added to each well. Then samples, control, and standards were loaded into the same wells. Incubation for 2 h was done, followed by 4 washing steps. Then, 100 μl of Rat IL6 conjugate was added to each well, the plate was then incubated for 2 h. The washing step was repeated and 100 μl of substrate solution was added and left for 30 min. Finally, 100 μl of stop solution was added to each well and optical density was obtained at 450 nm.

### Cell ROS

CellROX^TM^ Orange Reagent (Thermo Fisher Scientific C10443) was used for oxidative stress detection. The experiment was conducted according to the manufacturer’s instructions. Here, 5 μM dilution of the stain was prepared and added to the cell’s pallets followed by 30 min incubation at 37°C. After that, cells were washed three times with PBS and resuspended in 1 ml PBS. Then, Tali^TM^ slide reader was used to read fluorescently stained vs. unstained cells.

### Statistical Analysis

Distribution was determined using the D’Agostino-Pearson normality test. Parametric data were analyzed using one way-ANOVA with Sidak *post-hoc* for multiple comparisons test while non-parametric data were analyzed using the Kruskal-Wallis test with Dunn’s *post-hoc* test. In all analyses, a *p*-value of less than 0.05 was considered statistically significant.

## Results

### Synthesis and Characterization of SPIONS

**FIGURE 2:**
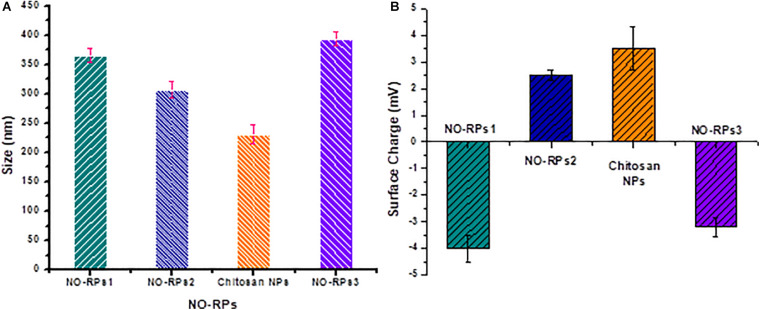
Characterization of the developed SPIONS and NO-RPs3. **(A)** TEM images of the prepared SPIONS (dark areas represent SPIONS), the selected area is an electron diffraction (SAED) image (inset). **(B)** Vibrating sample magnetometer (VSM) results of SPIONS.

Superparamagnetism is a magnetism that occurs particularly within a specific size range (3–50 nm) depending on the type of used materials. For magnetite, for instance, the size of the nanoparticles should be below 25 nm (Baumgartner et al., 2013), where the particles act as one single domain with higher susceptibility for magnetism as compared with their corresponding paramagnetic materials. In the case of using such magnetic materials in drug delivery, an additional characteristic of superparamagnetic materials must be considered. Furthermore, SPIONS as magnetic nanoparticles have been reported as promising materials for cell and bio-imaging during drug delivery applications. Drug carriers incorporating magnetic nanoparticles have been used as potential materials for theranostics (Singh et al., 2014a). This characteristic is the instantaneous turning of the magnetization back to zero when the external magnetic field is switched off with negligible remanence and coercivity, and hence agglomeration and possible capillary vessels embolization are evaded (Manuel et al., 2007; Singh et al., 2012, 2014b). In the current study, ethanolamine was used during SPIONS preparation for two main reasons that involve increasing the medium pH to form the corresponding oxides, and to act as a good capping agent for fulfilling the very small size needed as well as stabilizing the formed SPIONS. The size and morphology of the developed SPIONS were determined using HR-TEM imaging as in Figure 2A. As apparent from the figure, the developed SPIONS are spherical with a size around 12 nm as an average. The selected area electron diffraction (SAED) image in the figure inset, demonstrates the crystalline nature of the obtained SPIONS. The magnetic characteristics were measured at room temperature with the aid of VSM and illustrated by the hysteresis loops as in Figure 2B, which demonstrated good magnetism with a specific saturation magnetization (Ms) at about 63.5 emu/g. These good results can be returned to the small size of the prepared SPIONS, which decreases the oxygen content and increases iron content, as reported earlier by Thapa et al. (2004).

### Preparation and Characterization of NO-RPs

**FIGURE 3:**
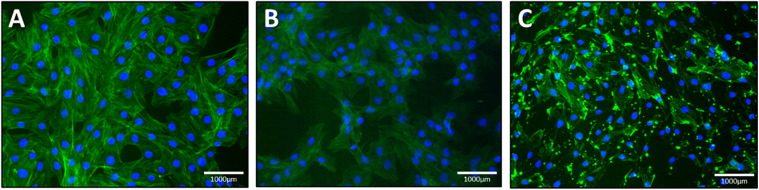
Morphological investigation using SEM for. **(A)** NO-RPs1 formula. **(B)** NO-RPs3 formula. **(C)** Chitosan nanoparticles incorporating L-Cysteine.

NO-RPs were prepared for delivering and releasing NO gas in a sustained and controlled manner. Polymeric nanocarriers have proven to be efficient in developing such nanoparticles due to their high biocompatibility, biodegradability in addition to being easily surface modified. The unique advantage of these formulas is that they could overcome the limitations of the systemic NO donors that include the short lifetime of the released NO gas and rapid metabolization of cGMP by phosphodiesterase (types I and V) enzymes (El-Sherbiny et al., 2015). Morphological investigation using SEM showed the successful formation of spherical-shaped nanoparticles for NO-RPs and chitosan nanoparticles incorporating the reducing agent as shown in Figure 3.

**FIGURE 4:**
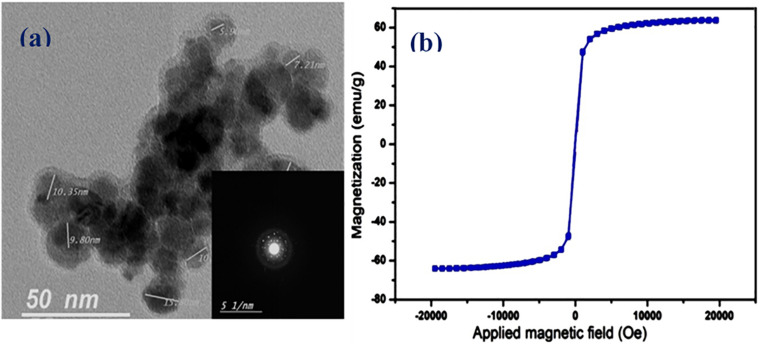
Zeta size measurement for the different prepared nanoparticles. **(a)** Size of nanoparticles. **(b)** Zeta potential of nanoparticles.

The size and surface charges of each of the prepared nanoformulations were assessed precisely using the Zetasizer as shown in Figures 4A,B, respectively. It was observed as illustrated in Figure 4A that NO-RPs3 possessed the largest size (393 ± 10 nm) as it incorporates SPIONS (10% w/w), the NO-precursor salt and the reducing agent, followed by the NO-RPs1 which demonstrated an average size of 365 ± 12 nm, as it incorporates both the precursor and the reducing agent within its structure, then followed by NO-RPs2 (306 ± 14 nm) that incorporates only the precursor. Finally, the chitosan nanoparticles incorporating the reducing agent showed the smallest size among all the formulas. The surface charge assessment in Figure 4B was found relevant to the composition of each of the tested nanoparticles. For instance, both NO-RPs1 and NO-RPs3 formulations depicted negative charges. This may be attributed to the high abundance of L-cysteine, the reducing agent in these two formulations. On the other hand, NO-RPs2 which possesses only the precursor demonstrated positively charged particles. Although the chitosan nanoparticles incorporated the L-cysteine reducing agent, they showed a net positive charge. This reflects the presence of the highly positive charged chitosan in a ratio of 2:1 compared to the incorporated cysteine. Consequently, the high positive charges of chitosan were sufficient to neutralize the negative charges of cysteine and to impart a net positive charge for the entire nanoparticles. The presence of high charges on the particle surface represents an advantage as they induce Columbic forces between the nanoparticles that overcome the Vander der Waals attraction among them. This results in preventing the aggregation of these particles in the suspension thus facilitating their dispersion in an aqueous medium to form highly stable colloidal nanosuspension (Ali et al., 2016; Khalil et al., 2019).

### NO Release Pharmacokinetics From the Developed NO-RPs

**FIGURE 5:**
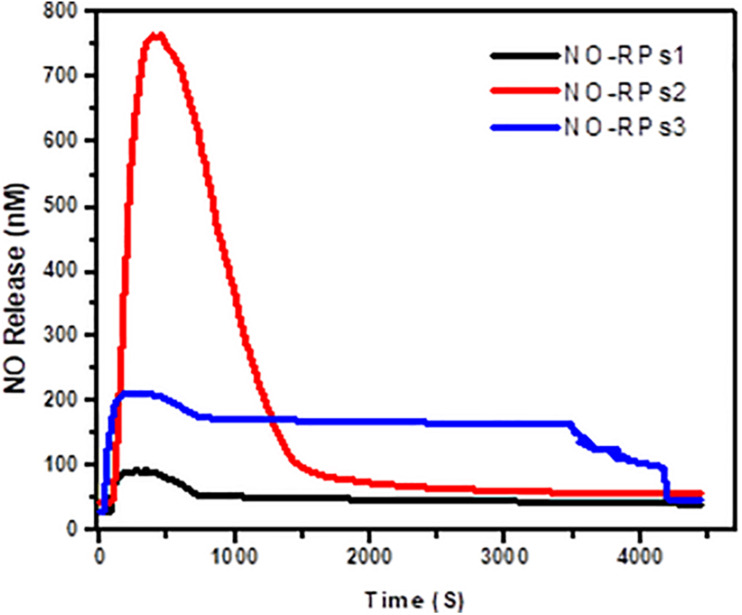
Estimation of NO release pharmacokinetics from the developed NO-RPs nanoformulations using NO electrode.

NO electrode was used to detect the pharmacokinetics of NO release from the different developed NO-RPs. A concentration of 5 mg/ml from each solution was estimated individually. It was found, as shown in Figure 5, that NO-RPs2 has an intensely higher initial NO release profile than NO-RPs1 and NO-RPs3 followed by a sustained release profile. It is observed that the initial amount of NO released from NO-RPs2 exceeds 7 times and almost 3 times the amount released from NO-RPs1 and NO-RPs3, respectively. It could be also noted from the figure that both NO-RPs1 and NO-RPs3 attained a more sustained release profile of NO gas with NO-RPs3 attaining almost double the amount of NO released at any time, which may be attributed to the presence of SPIONS within the NO-RPs3 structure. These SPIONS may play a catalytic role and accelerating the interaction between both the NO-precursor and the reducing agent.

### Viability Results Following Bubble Induced Shear Stress Exposure in the Flow Chamber

**FIGURE 6:**
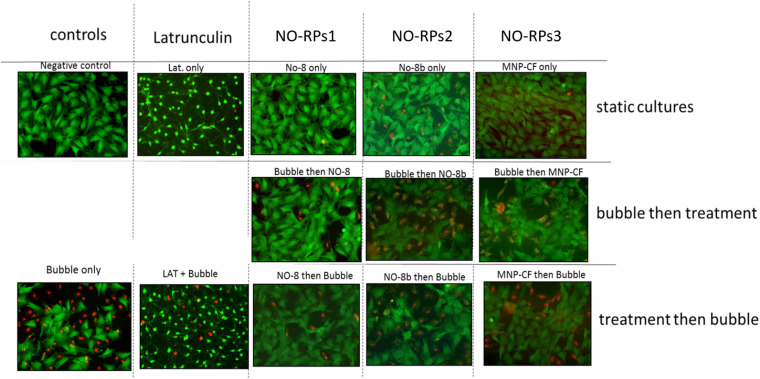
Live/dead staining results for the three nanoparticles tested on 149 lung epithelial cells. Upper column is for static cultures, middle column is for treatment experiments followed by the bubble and lower column is for bubble experiments followed by the treatment. NO-RPs1 enhanced viability most efficiently.

**FIGURE 7:**
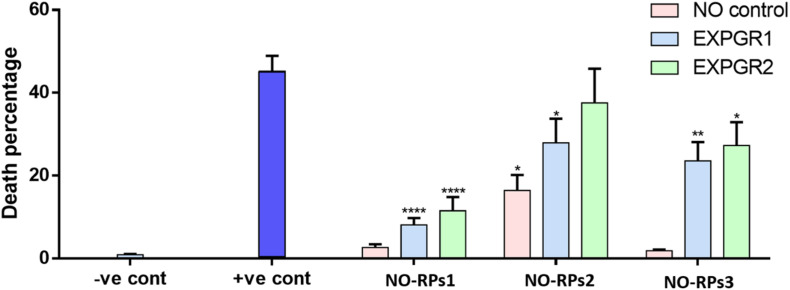
Bar chart for the three nanoparticles tested on 149 L2 cells. Showing significant decrement on death percentage when using NO-8 and MNP-CF nanoparticles after and before applying shear stress.

Here, we exposed confluent cell monolayers to bubble induced stresses. In this project, we investigated the effects of keeping the cells in three types of NO-RPs before or after cell exposure to bubble propagations. As mentioned, we designed 7 groups and for each group, we repeated the experiments at least 6 times. Figure 6 shows representative fluorescent pictures taken for each experimental group and Figure 7 shows death percentages for each experimental group in bar chart representation, as shown keeping the cell in serum-free media with NO solution did not lead to cell death for NO-RPs1 and NO-RPs3 nanoparticles, but there is an insignificant cell death for NO-RPs2 nanoparticles (∼15%). This suggests that instant high NO exposure may have a slightly detrimental effect on cells. Bubble flows resulted in significant cell injury with an average of ∼43%. NO exposure before or after to bubble flows decreased cell death significantly to ∼8 and ∼10% for NO-RP1nanoparticles and ∼23 and ∼27% for NO-RP3 nanoparticles. NO-RPs2 nanoparticles showed a slight decrease in cell death when administrated before bubble propagation. However, NO-RPs2 showed insignificant results when compared to positive control when exposed after bubble propagation.

### Actin Polymerization/Depolymerization Assessment

**FIGURE 8:**
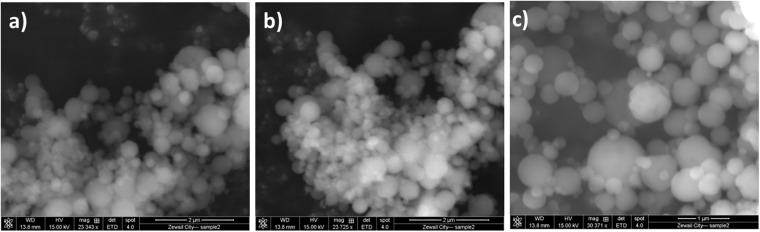
Lung epithelial cells stained with cytoskeleton staining. **(a)** control, **(b)** NO treated cells and **(c)** latrunculin treated cells. Green represents the actin stained cells whereas the blue is DAPI labeled nuclei.

We stained 149 L2 cells with cytoskeleton staining using actin labeled phalloidin. Cells treated with NO were compared to cells treated with latrunculin (positive control) as latrunculin is known to induce cell depolymerization. Figure 8 represents the cytoskeleton for latrunculin and NO treated cells compared to control (maintained in cell media only).

### AFM and Cell Morphology

**FIGURE 9:**
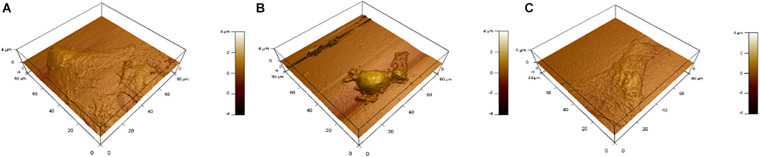
Topography via AFM. **(A)** Control, **(B)** latrunculin, and **(C)** NO treated.

Figure 9 shows topography maps obtained via AFM. Both latrunculin and NO caused shrinkage of the cells. This shrinkage and change in morphology indicate changes in the cytoskeleton which maintain cellular shape and rigidity.

### Quantitative RT-PCR (qRT-PCR)

**FIGURE 10:**
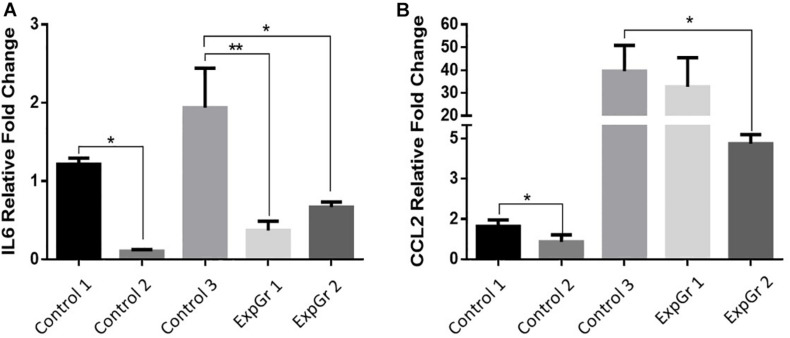
Relative gene expression of inflammatory markers. Lung epithelial cells gene expression of **(A)**. Interleukin 6 (IL6), and **(B)** chemokine (C-C motif) ligand 2 (CCL2) was assessed via qRT-PCR. Cells were cultured until 80% confluence then exposed to either normal media (Control 1), nitric oxide nanoparticles (Control 2), shear stress (Control 3), nitric oxide nanoparticle then shear stress (ExpGr 1), and shear stress then nitric oxide nanoparticles (ExpGr2). Cells were then collected for RNA isolation, cDNA preparation and qRT-PCR analysis. The analysis was by one-way-ANOVA with Sidak *post hoc* test for multiple comparison between the different groups. Data is presented as mean ± SEM. *N* = 4 for all groups. *Significant; **Highly significant.

As NO-RPs1 showed the best cellular recovery, therefore, we performed qRT-PCR for this group. qRT-PCR was done for cells treated with NO-RPS1 before or after exposure to shear to evaluate the gene expression changes. For that, β-ACTIN was chosen as a reference gene to normalize the expression of the genes of interest. Cumulative threshold values showed that the β-ACTIN gene expression is stable across studied groups (Supplementary Figure 1). As shown in Figure 10, Treatment with NO pre and post-exposure to shear (ExpGr 1 and ExpGr 2, respectively) caused a significant decrease in IL6 (*p* = 0.005 and 0.034, respectively). In the case of CLL2, only ExpGr 2 showed a significant reduction in the gene expression level (*p* = 0.024).

### IL-6 Protein Expression

**FIGURE 11:**
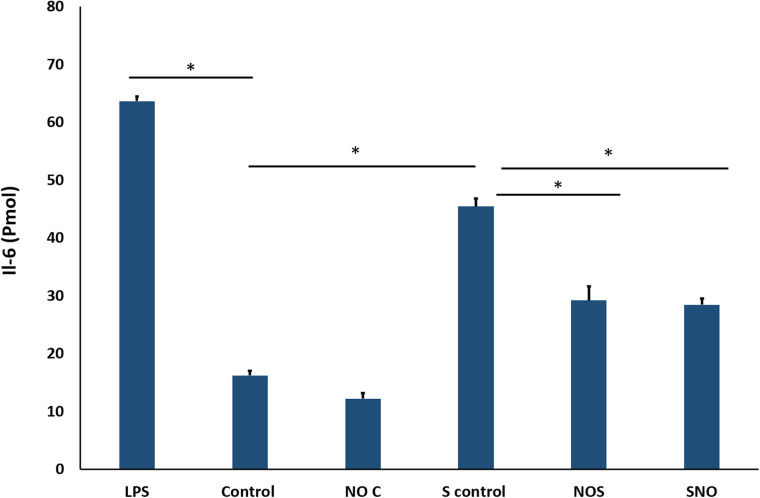
ELISA result for IL-6. The signal is reported as the total detected signal in (Pmol) divided by the cell count for each condition. NOC Analysis was by one-way-ANOVA with Sidak *post hoc* test for multiple comparisons between the group. Data is presented as mean ± SEM. *N* = 4 for all groups. *Significant.

To confirm PCR results, IL6 protein secretion into the media was measured using ELISA. The standard curve showed an R^2^ of 0.9994 (Supplementary Figure 2), which makes it acceptable for sample concentration determination. Here, LPS was used as a positive control to induce inflammation in cells. Treatment with NO pre-exposure to shear stress showed a significant reduction in the IL6 secretion (*p* = 0.0057, Figure 11).

### Cell ROS

**FIGURE 12:**
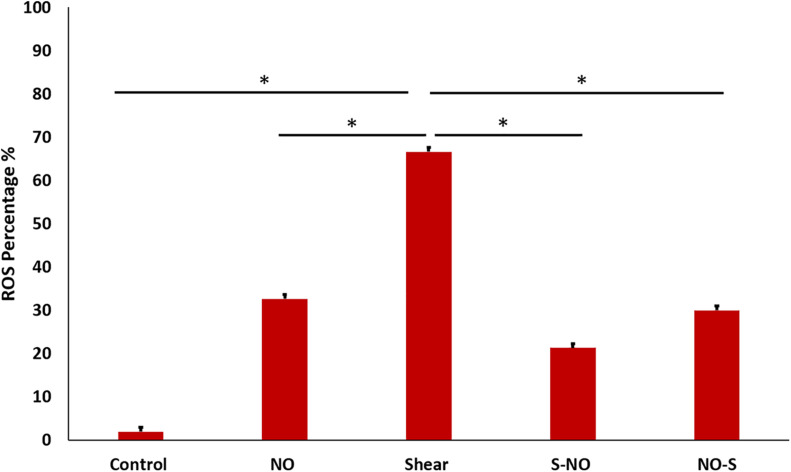
ROS percentage for different groups, showing significantly high ROS value for shear control, which becomes less after and before NO treatment.

**FIGURE 13:**
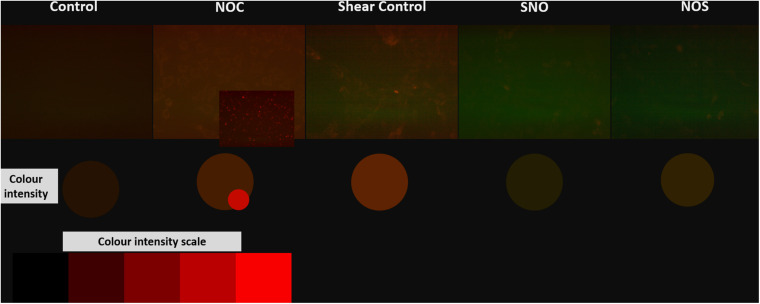
Microscopic images for different groups stained with orange cell ROX stain. The color intensity was compared to the red color intensity saturation scale.

Cell ROS was done to assess oxidative stress in different groups. As expected from previous tests (PCR and ELISA) ROS level was the highest in the shear control group which significantly decreased before and after NO exposure. The NO control group showed a slightly higher ROS level compared to the untreated control (Figure 12). Microscopic images for all groups were taken, the microscopic results were similar to those obtained from the Tali slide reader (Figure 13).

## Discussion

ARDS is mainly managed by mechanical ventilators therapy that involves inducing positive pressure to the lungs (Fan et al., 2018). However, VILI can be induced by mechanical forces from mechanical ventilation, which leads to further injury to the already diseased part (Biehl et al., 2013). Recently, many efforts have been made to decrease VILI associated with ARDS. NO therapy was shown to be effective in enhancing cell viability, nevertheless, it is limited to its short half-life (Thomas, 2001). NPS loaded with NO can overcome the half-life problem. In this study, three different NO nanoparticles were produced with different compositions.

Here, we showed that NO-RPs1 and NO-RPs3 save the cells both before and after injury. NO-RPs1 showed the best results in protecting the lung cells. The results suggested more recovery when NO was applied before the injury. We tried to understand the mechanism by which NO loaded NPs protects the cells from injury. This was attributed to two main mechanisms: cytoskeleton remodeling and reducing stress-induced inflammation. Depolymerization of the actin cytoskeleton and softening in endothelial cells were shown to be directly correlated with NO synthesis (Versari et al., 2007; Fels et al., 2014). This supports our data for cytoskeleton staining and AFM analysis, where actin filaments appear faint compared to the control group. This recommends actin filaments depolarization. Cell cytoskeleton softening was shown to protect the cells against injuries (Yalcin et al., 2018). As NO treatment could depolymerize the cell’s cytoskeleton before the injury, more cells are recovered compared to NO treatment after injury, which suggests that the main mechanism by which NO protects cells is by cytoskeleton remolding. Reducing inflammation is another mechanism, but its role is less effective than manipulating the cytoskeletal filaments. As the results suggest reduction in IL-6 in both experimental groups without significant difference between the two groups, which explains NO has an anti-inflammatory role, however, it’s not sufficient to fully protect all injured lung cells. The anti-inflammatory role of NO was attributed in literature due to its prevention of cytokines storms and enhancing the oxygenation (Van Der Vliet et al., 2000; Adusumilli et al., 2020).

From the three particles, only NO-PRs1 showed an effective role in protecting lung cells after injury, although it has the lowest NO release compared to the other two particles. This suggests that the NO-delivered by NO-PRs1 is enough to protect the cells. The further increment will either have a less efficient effect or toxic effect as in NO-PRs2 (Akmal and Hasan, 2008). This might be mainly due to the formation of oxidant peroxynitrite at high NO concentrations, which induces DNA damage and cell death (Van Der Vliet et al., 2000). Furthermore, at high concentrations, NO acts as a pro-inflammatory mediator (Sharma et al., 2007). This suggests that the best NO effect is when it is released in low concentrations at a steady rate.

There has been a major effort in recent years to reduce VILI associated with ARDS. Most studies focused on decreasing mechanical stresses during ventilation to decrease cellular injuries. Using an *in-vitro* system of airway reopening, we have exposed epithelial cell monolayers to bubble propagation and showed that decreasing flow-induced stresses over epithelial cells enhances cell viabilities (Dailey et al., 2007; Yalcin et al., 2007, 2009). As a result of similar efforts (Higuita-castro et al., 2014), safer ventilation strategies were adopted clinically. For example, high-frequency oscillatory ventilation (i.e., application of frequencies greater than 60 breaths per minute, 1 Hz, to deliver small tidal volumes) was shown to enhance oxygenation and decrease VILI in ARDS patients and is now a common clinical practice (Meyers et al., 2019). More recently, an alternative approach was developed by us and others to decrease cellular susceptibility to mechanical stresses of VILI. In this approach, cell mechanics are altered before exposure to flow-induced stresses during airway reopening. Using our *in-vitro* airway reopening model, we showed that, disruption of the cytoskeleton with Latrunculin before bubble exposure, dramatically decreases cell injuries (Yalcin et al., 2018). Cell mechanics measurements revealed, Latrunculin treated cells became more viscous, in other words, more “fluid-like” suggesting that flow-induced forces were dampened and did not cause deformation and rupture of the cell membrane. More recently, using a similar *in-vitro* system, a clinical Statin, Simvastatin was shown to have a beneficial effect in epithelial cell survival under flow-induced stresses, through depolymerization of the actin cytoskeleton and decrease in cell elasticity (Astro et al., 2016). These findings provided evidence that effective delivery of clinical agents into the deep lung, which can decrease inflammation and alter cell mechanics will have substantial benefits to decrease VILI. This is particularly useful to decrease mortalities in severe infections for SARS-CoV-2. Additionally, the ROS level was reduced when treated with NO both before and after bubble propagation, which is mainly due to the interplay role of NO and ROS. As it has been reported that NO and ROS interactions are complex, it can be synergistic, antagonistic or they work in parallel (Lindermayr and Durner, 2015). In our case, ROS was reduced, and cell survival was enhanced. Although the ROS value obtained from Tali slide reader in NO treated group showed a slightly higher value than the control (no significant difference), the microscopic image showed stained particles at a different focus. This can explain the slightly higher value of ROS from Tali reader, that the unspecific staining of the particles contributed to the high value.

## Conclusion

Delivering NO particles with the aid of biotechnology might be an effective treatment for ARDS, considering the positive effects of increasing softness and reducing inflammatory markers. NO must be delivered at low concentrations with a steady rate, otherwise, a toxic effect might be induced.

## Data Availability Statement

The original contributions presented in the study are included in the article/Supplementary Material, further inquiries can be directed to the corresponding author/s.

## Author Contributions

SS: drafting the manuscript, conduction of experiments, and revision. IE-S: conception or design of the work and drafting. MA: conduction of experiments and revision. IA, HK, and AA-S: conduction of experiments. AP: AFM part. FB: statistical analysis and revision. HY: conception and design of the work, critical revision, and final approval for the manuscript to be published. All authors contributed to the article and approved the submitted version.

## Conflict of Interest

The authors declare that the research was conducted in the absence of any commercial or financial relationships that could be construed as a potential conflict of interest.
