# Supplementary material for: Nitric Oxide Releasing Hydrogel Nanoparticles Decreases Epithelial Cell Injuries Associated With Airway Reopening
Source: Front Bioeng Biotechnol. 2021 Jan 5;8:579788. doi: 10.3389/fbioe.2020.579788 (PMC7813943; doi:10.3389/fbioe.2020.579788)
Supplement: Supplementary file 1 [file Data_Sheet_1.docx]

Supplementary materials

**Nitric oxide Releasing Hydrogel Nanoparticles Decreases Epithelial cell Injuries Associated with Airway Reopening**

Samar Shurbaji, Ibrahim M. El-Sherbiny, Isra H. Ali2, Haya and Ameena, Maha Alser, Fatiha Benslimane, Anton Popelka, Magdi Yacob, Huseyin C Yalcin

**S1: Supplementary materials and methods:**

**Relative gene expression of inflammatory markers:**

To verify the effect of NO on cells, qRT-qPCR was done to compare the relative gene expression level of different inflammatory factors released from rat EpC (IL-6, CCL-2 and CXCL-2). 3 independent experiment were conducted on each ExpGr.

**RNA extraction:** First, total RNA was extracted from the cells using TriZol reagent. First, 500 µl of TriZol reagent were added gradually on the treated area of cultured L2 (ATCC® CCL­149™) cells and vigorously mixed by the pipette and transferred to a clean Eppendorf tube. Then, the lysate was incubated for 5 minutes at room temperature followed by centrifugation for 10 minutes at 12,000g. The supernatant was transferred to a new tube followed by adding 50 µl of chloroform and shaking. The sample was left standing for 5 minutes followed by centrifugation for 5 minutes, at 12,000 g. the aqueous top layer was transferred to a new tube. Afterward, 250 µl of isopropanol was added to RNA and kept standing for 10 minutes. Centrifugation for 10 minutes at 12,000 g was done after that and the supernatant was removed. Finally, washing step was repeated twice by adding 500 µl of absolute ethanol, vortexed and centrifuged for 5 minutes at 12,000 g. The supernatant was completely removed, and the pellet was left to air dry (~15 minutes). The pellet was resuspended in 25 µl of Nuclease-free water and quantified using nanodrop-1000 spectrophotometer (Thermo Scientific, USA). Samples with 260/280 ratio between 1.8 - 2.1 were accepted as pure.

**cDNA synthesis:** second, the RNA samples were used as template RNA for reverse transcription and cDNA synthesis using the SuperScript IV VILO Master Mix kit (Invitrogen).. 300 ng of RNA were added to 8 µl of master mix and the reaction volume was completed to 20 µl with nuclease-free water. The reaction took place in the thermal cycler (GeneAmp PCR system 9700, Applied Biosystem). the instrument was programmed as: 25°C for 10 minutes, 50°C for 10 minutes, 85°C for 5 minutes, and 4°C until the samples were picked. All samples were kept in the -20°C freezer until use.

**qRT-PCR:** qRT-PCR was performed in 3 technical replicates for the 3 genes using SYBR Green master mix (applied biosystems) as suggested by manufacturer. The reaction was run in a 96-well plate. Each well had a total reaction volume of 12 µl, well, (6 µl of SYBR green master mix, 0.6 µl of each primer (1mM), 2.8 µl nuclease-free water, and 2 µl of cDNA sample). The plate was sealed, centrifuged, and the reaction was run. The expression levels were normalized to the beta ACTIN reference gene. The relative quantity was calculated using ∆C_T_ method described by ([Schmittgen and Livak, 2008](#_ENREF_37)).

**S2. supplementary results:**

**Supplementary figure 1: Reference gene (b actin) CT values is stable between the different treatments**. Cells were cultured until 80% confluence then exposed to either normal media as control (C), nitric oxide nanoparticles (NOC), shear stress (SC), nitric oxide nanoparticle then shear stress (NOS), or shear stress then nitric oxide nanoparticles (SNO). Cells were then collected for RNA isolation, cDNA preparation and QPCR analysis. No difference was observed in the expression of β actin between the studied groups. Analysis was by one-way-ANOVA with Sidak post hoc test for multiple comparison between the groups; C vs NOC, C vs SC, SC vs NOS and SC vs SNO. Data is presented as mean ± SEM. N=4 for all groups.

**Supplementary figure 2: ELISA standard curve.** A curve fit was generated from IL6 standards and the mean absorbance measured at 450 on the y-axis against its corresponding concertation on the x-axis. Linear regression was performed to linearize the data and generate equation for sample concertation determination
